# Wry-Neck

**Published:** 1894-01-13

**Authors:** 


### ROYAL ORTHOPÆDIC HOSPITAL.

### Wry-Neck.

This deformity may be caused in many ways. It is often met with as the result of spastic, or permanent contraction of the sterno-mastoid or some other of the rotatory muscles ofthe neck. In other cases it is produced by the contraction of scars, resulting either from inflammation of the deep tissues, or burning of the skin. The most common variety and the one most amenable to treatment is that which is known as congenital wry-neck. In this the sterno-mastoid muscle on the affected side is contracted, and may even be replaced by a hard rounded cord, due to its almost complete destruction.

The position of the patient is then characteristic. The neck is twisted, so that the face is turned towards the unaffected side, and it is flexed so that the head tends to lie on the shoulder of the affected side. If the case comes under treatment early this is all that is found, but if it has existed some years, the spine becomes affected and lateral curvature is the result. It is, therefore, considered well to treat the case as early in life as it shows itself in order to prevent this undesirable addition to its difficulties.

The cause of congenital wry-neck is obscure. The attempt has repeatedly been made to show that it results from violence during birth. This possibly accounts for some of the cases in which shortly after birth a swelling has been found in the sterno-mastoid muscles, but in others the deformity is found, although there was no birth injury to cause it, and no swelling of the sterno-mastoid that might suggest rupture of any of its fibres. At the Royal Orthopædic Hospital these cases are treated by division of the contracted muscle. The operation is performed in this way: Chloroform is administered, and the shoulder is raised with the head drawn over by an assistant in such a way as to stretch the muscle. A tenotomy knife is inserted behind the tendon close above the collar-bone. Should the tendon be wide the knife is inserted between its two heads, which are then divided backwards or forwards. After division of the bands the puncture is closed with a piece of lint, and for a day or two no active treatment is pursued.

If the case be slight it is not considered necessary to order any instrument, but the head is moved night and morning in such a way as to thoroughly stretch the affected muscle. In more severe cases an instrument is applied. This consists essentially of a strap round the head, to which a band is fastened. This band in turn is fixed to a moulded leather belt round the chest. By adjusting the apparatus the head can be drawn over so as to stretch the sterno-mastoid muscle. Some surgeons, again, use a sort of necklet of leather, which rests on the shoulders and supports the head. It is made of stiffened leather, and is so shaped as to press especially on the affected side of the head. In very severe cases a stronger instrument with a screw action is used.

Whatever be the nature of the instrument that is employed, great care is always taken to move the head, and cause it to be moved by the patient several times a day in such a way as to stretch the sterno-mastoid and prevent its recontraction.

If it is found that there is any curvature of the spine it is treated by the ordinary spinal apparatus. In those cases in which the face is deformed, and that is to say in all in which the wry-neck has been permitted to remain for some years, no treatment can give an absolutely satisfactory result, since it is impossible to correct the form of the face which does not develop properly, and so gets a lop-sided appearance, one side being smaller and lower down than the other. Even in these cases, however, considerable good can generally be done by the treatment above described.

